# Wearable, Multimodal, Biosignal Acquisition System for Potential Critical and Emergency Applications

**DOI:** 10.1155/2021/9954669

**Published:** 2021-06-10

**Authors:** Chin-Teng Lin, Chen-Yu Wang, Kuan-Chih Huang, Shi-Jinn Horng, Lun-De Liao

**Affiliations:** ^1^Institute of Electrical Control Engineering, National Yang Ming Chiao Tung University, Hsinchu, Taiwan; ^2^Brain Research Center, National Yang Ming Chiao Tung University, Hsinchu, Taiwan; ^3^Australia Artificial Intelligence Institute, Faculty of Engineering and Information Technology, University of Technology, Sydney, NSW 2007, Australia; ^4^Department of Computer Science and Information Engineering, National Taiwan University of Science and Technology, Taipei, Taiwan; ^5^Institute of Biomedical Engineering and Nanomedicine, National Health Research Institutes, Miaoli County, Taiwan

## Abstract

For emergency or intensive-care units (ICUs), patients with unclear consciousness or unstable hemodynamics often require aggressive monitoring by multiple monitors. Complicated pipelines or lines increase the burden on patients and inconvenience for medical personnel. Currently, many commercial devices provide related functionalities. However, most devices measure only one biological signal, which can increase the budget for users and cause difficulty in remote integration. In this study, we develop a wearable device that integrates electrocardiography (ECG), electroencephalography (EEG), and blood oxygen machines for medical applications with the hope that it can be applied in the future. We develop an integrated multiple-biosignal recording system based on a modular design. The developed system monitors and records EEG, ECG, and peripheral oxygen saturation (SpO_2_) signals for health purposes simultaneously in a single setting. We use a logic level converter to connect the developed EEG module (BR8), ECG module, and SpO_2_ module to a microcontroller (Arduino). The modular data are then smoothly encoded and decoded through consistent overhead byte stuffing (COBS). This developed system has passed simulation tests and exhibited proper functioning of all modules and subsystems. In the future, the functionalities of the proposed system can be expanded with additional modules to support various emergency or ICU applications.

## 1. Introduction

The monitoring of biosignals is becoming increasingly important in modern healthcare and health management, especially for emergency or intensive-care units (ICUs) [1–3]. Patients with unclear consciousness or unstable hemodynamics often require aggressive monitoring by using multiple monitors. This type of monitoring is not only used to understand current physiological changes in the body but also provides a potential way to predict the occurrence of specific malfunctions (e.g., stroke or heart attack) [4]. Biosignals record a biological event (such as a beating heart or a contracting muscle) in both space and time. The electrical, chemical, and mechanical activities that occur during this biological event often produce signals that can be measured and analyzed [5]. Biosignals, therefore, contain useful information that can be used to understand the underlying physiological mechanisms of a specific biological event or system and may be useful for medical diagnosis [6, 7]. These signals can be acquired in a variety of ways [5, 6, 8]. Many developed products have combined different peripheral measurements (heart rate (HR), skin conductance level, etc.) into one actigraphy for healthcare [9, 10]. However, very few studies or products have combined these peripheral activities with central biological changes, such as brain activities (electroencephalography (EEG), hemodynamics, etc.) [11–13].

Moreover, recent developments have shown that multiple biological signals can provide higher accuracy in terms of determining human performance [14, 15] or diagnosing various diseases [16]. Biological signals in living beings are any signal (either electrical or nonelectrical) that can be continually measured and monitored to provide information about several different systems in the body [17]. For example, EEG can be used to assess brain function diseases, degenerative diseases, inflammation, epilepsy, and abnormal sleep [18–22].

In addition, electrocardiography (ECG) can be used to observe cardiac behavior and to detect arrhythmia, ventricular atrial hypertrophy, myocardial infarction, myocardial ischemia and other diseases [23]. It can reflect the degree of myocardial damage and the structure of the atria and ventricles [24, 25]. Recently, oxygen saturation (SpO_2_) combined with ECG was used to detect stenosis or occlusion of an arteriovenous fistula/graft, the progression of arteriosclerosis, and arrhythmia [25]. SpO_2_ is a measure of the amount of oxygen-carrying hemoglobin in the blood relative to the amount of hemoglobin not carrying oxygen [26–29]. The body requires a certain level of oxygen in the blood to function efficiently [26, 28, 30]. In fact, very low levels of SpO_2_ can result in severe symptoms [26]. An ECG conditioning circuit is employed to examine the measured ECG signal. A pulse oximeter with a finger-type probe is connected to indirectly measure the SpO_2_ level, and then, the HR and SpO_2_ level are obtained at the same time [31, 32].

Therefore, an urgent need exists to develop an integrated system that can record multiple biosignals simultaneously for medical applications. In this study, we develop a multibiological integrated monitoring system that can acquire and process multiple biosignals (such as EEG, ECG, SpO_2_, and HR signals) simultaneously. Additionally, the developed system can transmit integrated biological data wirelessly to a smartphone or PC and monitor EEG, ECG, SpO_2,_ and HR information in real time. Most conventional systems take data points separately and from different locations on the body. Additionally, complicated pipelines or lines increase the burden on patients and inconvenience for medical personnel. In contrast, we develop an intelligent sensor system that facilitates sensor fusion by sealing the electronics into a single mold. This design is less affected by motion artifacts. In the future, this option will provide flexibility to patients and give clinicians access to physiological data in real time. Moreover, the modular design concept (functional partitioning into discrete and scalable modules) will soon be applied to this system to expand the number of possible measurements and establish industry standards.

## 2. Materials and Methods

### 2.1. System Architecture

**Figure 1:**
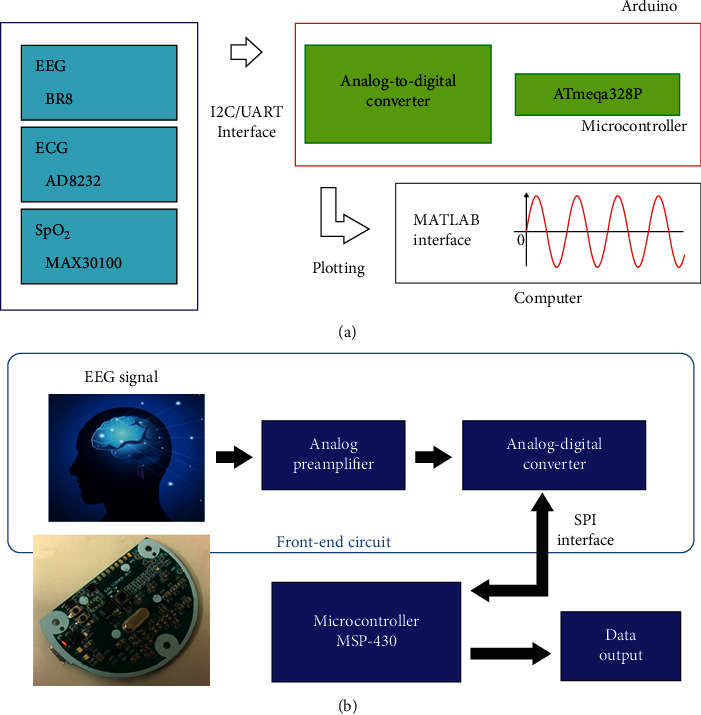
System architecture, including the EEG signal acquisition system. (a) System architecture. The system consists of three parts: recording modules, a microcontroller, and an online interface. The core system is the Arduino Mega 2560, which has the following characteristics: (1) an open-source single-chip microcontroller that uses an Atmel AVR single chip, (2) open-source software, (3) a hardware platform built into a simple I/O interface panel, and (4) a development environment based on the C language. Biosignals are acquired by separate related modules: (1) an EEG module (BR8), (2) an ECG module (AD8232), and (3) an SpO_2_ module (MAX30100). (b) The EEG signals are first amplified by two analog amplifier circuits and then sent to the ADC (ADS1298) for sampling. The digital signals converted by the ADC are transmitted to the microcontroller unit (MSP430) via an SPI. After the signals are encoded by MSP430, the raw EEG data are sent to other receiver devices through RS-232 interfaces for further analysis or application.

The purpose of this study is to develop a biosignal recording system that can measure multiple biosignals synchronously (the system architecture is shown in Figure 1(a)). The system consists of three parts: a recording module, a microcontroller, and an online interface. The core system is the Arduino Mega 2560, which has the following characteristics: (1) an open-source single-chip microcontroller that uses an Atmel AVR single chip; (2) open-source software; (3) a hardware platform built into a simple output/input interface panel; and (4) the use of C language to develop the environment. The biosignals are acquired by separate related modules: (1) EEG module: BR8; (2) ECG module: AD8232; and (3) SpO_2_ module: MAX30100.

### 2.2. EEG Signal Acquisition System

The architecture of the EEG signal acquisition system is shown in Figure 1(b). The EEG signals are first amplified by two analog amplifier circuits and then sent to the analog-to-digital converter (ADC) (ADS1298) for sampling. The digital signals converted by the ADC are transmitted to the microcontroller unit (MSP430) via a serial peripheral interface (SPI). After the signals are encoded by MSP430, the EEG raw data are sent to other receiver devices through RS-232 for further analysis or application.

#### 2.2.1. Front-End Active Circuit

In the first stage, INA333 has a high input impedance (100 GΩ) and a high common-mode rejection ratio (CMRR) (100 dB). These factors make INA333 suitable for the modular design of the integrated system.

The transfer function of this design is as follows:

(1) $$\begin{array}{c} \text{Gain}=1+\left(\frac{100\text{k}\Omega }{R_{G}\Omega +\left(1/jw\times 47\mu \text{F}\right)}\right). \end{array}$$

**Figure 2:**
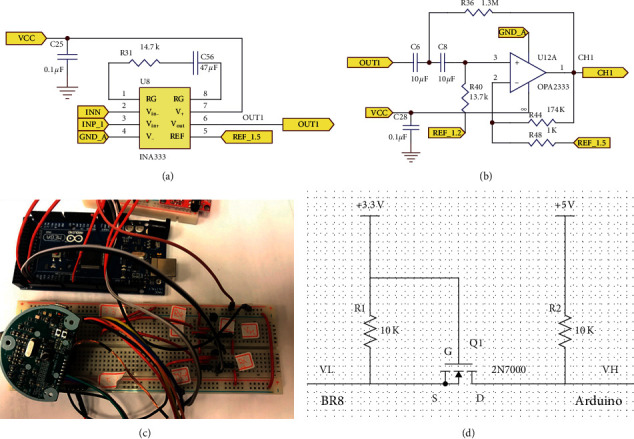
Front-end active circuit of the proposed system. Schematics of (a) the instrument amplifier and (b) the second-order high-pass filter. (c) Circuit of the logic level converter. Arduino systems generally work at 5 V, but the MSP430 used in our EEG system is a microcontroller operating at 3.3 V–3.7 V If a TX/RX is directly connected, it may easily cause the MSP430 system to wear out or even overheat. (d) Therefore, between the two systems, the logic level converter presented here is used for voltage conversion.

In this circuit, *R*_*G*_=14.7K is set as the gain of the amplifier. The preamplifier amplitude is set to 7.8 V/V, and the cutoff frequency is designed to be 0.103 Hz. Thus, the circuit achieves high performance in the first stage, as shown in Figure 2(a) (schematic of the instrument amplifier). In the second stage, OPA2333 has a low offset voltage (10 *µ*V), a low quiescent current (17 *μ*A), an excellent CMRR (130 *d*B), and a low power op amplifier. In this stage, OPA2333 is used as a bandpass filter. The second-order high-pass filter circuit is shown in Figure 2(b).

The EEG signal is filtered by a second-order high-pass filter with a cutoff frequency of 0.108 Hz in the second stage. The gain of the high-pass filter is set to 174 V/V. The transfer function of this designed high-pass filter circuit is as follows:

(2) $$\begin{array}{c} G\left(S\right)=\frac{121S^{2}}{S^{2}+1.11S+0.463},\\ S\approx 0\;\left(\text{low frequency}\right)\;Gain=\frac{0\text{V}}{\text{V}},\\ S\approx \infty \;\left(\text{high frequency}\right)\;Gain=\frac{174\text{V}}{\text{V}},\\ fL\;=\;0.108\text{Hz}. \end{array}$$

#### 2.2.2. Analog-to-Digital Converter (ADC)

In the ADC unit, ADS1298 integrates 8 channels of a high-resolution (24 bits) delta-sigma ADC and provides a high data rate (32 k samples per second). ADS1298 is usually used for ECG and EEG monitoring, as shown in Table 1.

Overall, the total gain of the active circuit is approximately 1,357 V/V, and the noise below 0.108 Hz is filtered out. The amplified and filtered EEG signals are digitized by the ADC unit (ADS1298). A total of 8 channel active circuits are operated at approximately 2.8 mA with a 3 V DC power supply. The details of the active circuits are shown in Table 1.

#### 2.2.3. Microcontroller: MSP430

After digitization is completed, the microcontroller unit (MSP430F5522, Texas Instruments, USA) receives the digital signals. MSP430 incorporates a 16-bit RISC CPU, peripherals, 10 KB SRAM, and 128 KB Flash. Dedicated embedded emulation logic resides on the device itself and is accessed via the Joint Test Action Group (JTAG) using no additional system resources. A sampling rate of 500 samples per second is set for the ADC.

MSP430 processes EEG signals and transmits them via a universal asynchronous receiver/transmitter (UART) interface to the Arduino board. Finally, the EEG data and other biosignals are displayed simultaneously on the screen and saved to the computer [8, 33].

### 2.3. ECG Module

AD8232 is a signal module for ECG measurement applications. It is designed to extract, amplify, and filter small biosignals under noisy conditions [34]. AD8232 is chosen because it has the best output impedance and gains. For the high-pass filter, a two-pole high-pass filter is used, and for the low-pass filter, a two-pole Sallen–Key low-pass filter is used [34]. AD8232 is available in a 4 mm × 4 mm, 20-lead LFCSP package. All the specifications make AD8232 suitable for use in the integrated system.

### 2.4. SpO_2_ Module

The SpO_2_ module used, MAX30100, is an integrated pulse oximetry and HR monitoring sensor. It combines two LEDs, a photodetector, optimized optics, and low-noise analog signal processing to detect pulse oximetry and HR signals. MAX30100 operates from 1.8 V to 3.3 V power supplies [35] and can simplify the circuit design, reduce the system footprint, and reduce the design time and system power consumption; hence, it is suitable for the system [35].

### 2.5. Microcontroller Unit

Arduino uses an open-source software and hardware platform and provides a simple I/O interface. It also supports development environments such as Java and C. In the experiment, we connected different modules to the Arduino Mega 2560. The Arduino board can accept input voltages from 7 to 12 V. In addition, it can output 3.3 V and 5 V to provide different physiological modules and has a sufficient number of I/O pins, making the integration of many physiological signal modules straightforward.

The Arduino Mega 2560 is an ATmega2560-based microcontroller board. It has 54 digital I/O pins (15 of which can be used as PWM outputs), 16 analog inputs, 4 UARTs (hardware serial ports), a 16 MHz crystal oscillator, a USB connection, and a power jack. It contains everything necessary to support the microcontroller and simply requires connection to a computer with a USB cable or boot-up with an AC-DC adapter or battery.

### 2.6. BR8 Connection to Arduino: Logic-Level Converter

The Arduino board generally functions at 5 V, but the MSP430 used in our EEG system is a microcontroller operating at 3.3–3.7 V. If the TX/RX is connected, it will easily wear out or even overheat the MSP430 system (Figure 2(c)). Between the two systems, a logic-level converter is used for voltage conversion (Figure 2(d)).

**Figure 3:**
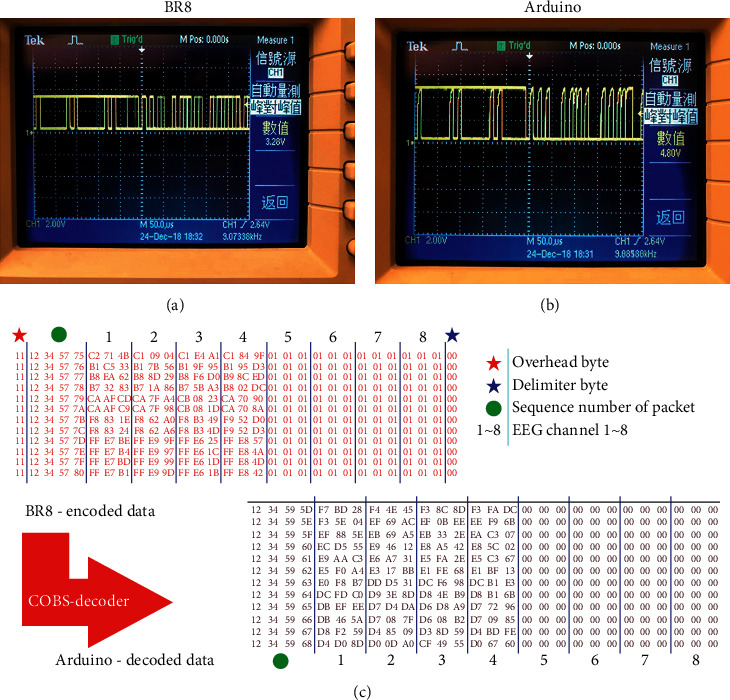
With the logic level converter, the transmitted peak-to-peak voltage of MSP430 (a) is converted from 3.28 V to 4.8 V as it becomes the received peak-to-peak voltage of the Arduino board (b). (c) Packets decoded by the COBS decoder.

The principle of this system is straightforward. When the current terminal (from the Arduino board) has a VL input of 5 V, the transistor does not conduct, and the back-end (to MSP430) VH output is 3.3 V. Conversely, when the front-end VL input is 0 V, the transistor is turned on, and the back-end VH output is also 0 V; thus, the voltage can be converted to a level suitable for different systems. Combined with the logic-level converter, the transmitting peak-to-peak voltage of MSP430 (Figure 3(a)) changes from 3.28 V to 4.8 V as it becomes the receiving peak-to-peak voltage of the Arduino board (Figure 3(b)). Additionally, the transmitting peak-to-peak voltage of the Arduino board changes from 4.8 V to 3.28 V as it becomes the receiving peak-to-peak voltage of MSP430.

Consistent overhead byte stuffing (COBS) is an algorithm for encoding digital packets to achieve efficient and reliable packet framing. It uses zero as a specific byte, which is inserted into a packet as a delimiter. When a zero data byte occurs, the operation replaces the zero bytes with a nonzero value. Therefore, zero data are not present in the packet and are misinterpreted as a packet boundary (shown in Table 2). This method enables the data packet receiver to quickly and efficiently restore the data packet to its original data form for efficient and error-free transmission.

The data transmitted from MSP430 to the Arduino board are encoded by using COBS. To integrate different signals from each module, the packets need to be decoded into the original data. After the packets are decoded, the overhead byte and the delimiter byte are eliminated, and the zero value is reduced, as shown in Figure 3(c).

## 3. Results and Discussion

### 3.1. EEG Test Result

The experiments reported in this paper were approved by the Institutional Review Board (IRB) of National Chiao Tung University (NCTU-REC-106-057) and followed the rules of the Declaration of Helsinki. In the EEG signal test, we use the formal EEG test signal generator to simulate the fixed frequency signal (500 *μ*V, 2 Hz sine wave) for testing. The basic signals are framed as a packet through the BR8 module and transmitted to the Arduino board. Then, the Arduino board restores the packet to the original waveform via a UART. The design of the proposed system successfully passes the EEG simulation test and obtains the same quality EEG signal as that obtained through the connection with the EEG module (BR8).

### 3.2. ECG Test Result

**Figure 4:**
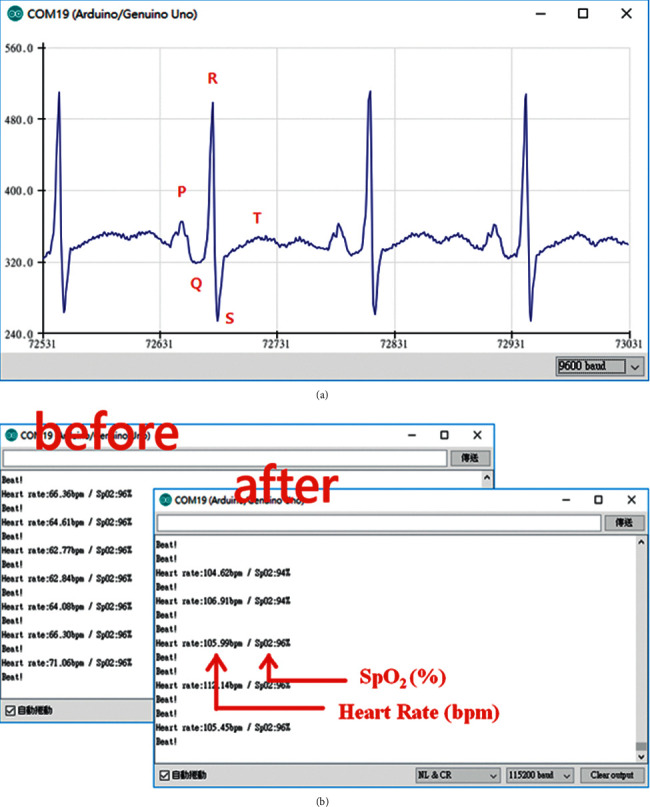
(a) Arduino interface and ECG measurement plot obtained by the Arduino device. (b) Blood oxygen measurement after climbing stairs.

In the ECG independent test, we use a UART to connect the AD8232 to the Arduino board through the TX/RX signal lines to receive the measured data from the experiments. The Arduino board then organizes the results into data for transmission to the computer as follows. The electrodes are grouped together to observe the activity of the heart from different angles. The typical ECG waveform can clearly be observed in Figure 4(a). PQRS and less obvious *T* waves can also be seen.

### 3.3. SpO_2_ Test Result

Unlike AD8232, MAX30100 uses I^2^C and the Arduino board to transfer data. Through the two signal lines SCL and SDA connected to the Arduino board, after the process starts, the red LED on MAX30100 is activated, indicating normal operation. The red LED plays an important role in measuring blood oxygen data.

When measuring, we press the index finger over the red LED, allowing light to pass through the finger, with the reflected rays being monitored by the receiver. The signal received by the receiver is analyzed with respect to both the AC signal and the DC signal. In addition to measuring the blood oxygen concentration, MAX30100 can calculate the rhythm.  Figure 4(b) shows the results of SpO2 transmission to the computer.

The data in the figure are updated approximately every second. We carry out a simple test: the subject is first measured in a calm state, and then, the subject climbs the stairs to the eighth floor and is measured again. A simple comparison reveals that the heart rhythm is significantly improved after climbing the stairs. The normal blood oxygen readings of an adult range from approximately 94% to 100%.

### 3.4. Graphical User Interface Test

To monitor a variety of biomedical signals, we must develop an integrated interface that can simultaneously display EEG, ECG, and SpO_2_ monitoring results. In this study, we use MATLAB to achieve our goal. MATLAB's powerful features provide us with a waveform interface and a collection of data packets from the COM port. The collected data are easy to store and use in subsequent calculations. In the experiment, we use the interface to display the EEG and ECG real-time waveforms, the blood oxygen level, and the HR. With MATLAB itself, we observe the individual waveform amplifications and store them at the end of measurement. The data for each experiment are available for later review.

### 3.5. Online Demo of the Integrating System

**Figure 5:**
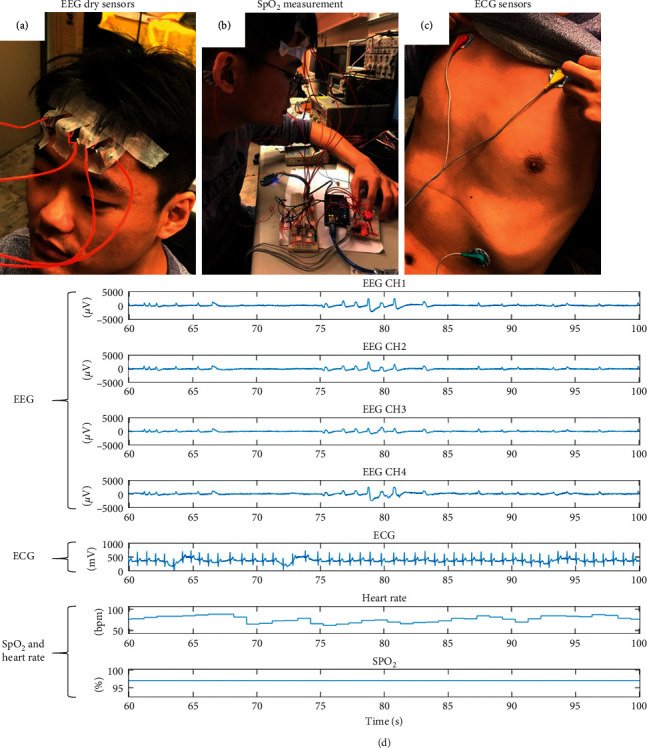
(a), (b), (c) Actual placement of the measurement modules. (d) Integrated measurement results for all signals.

**Figure 6:**
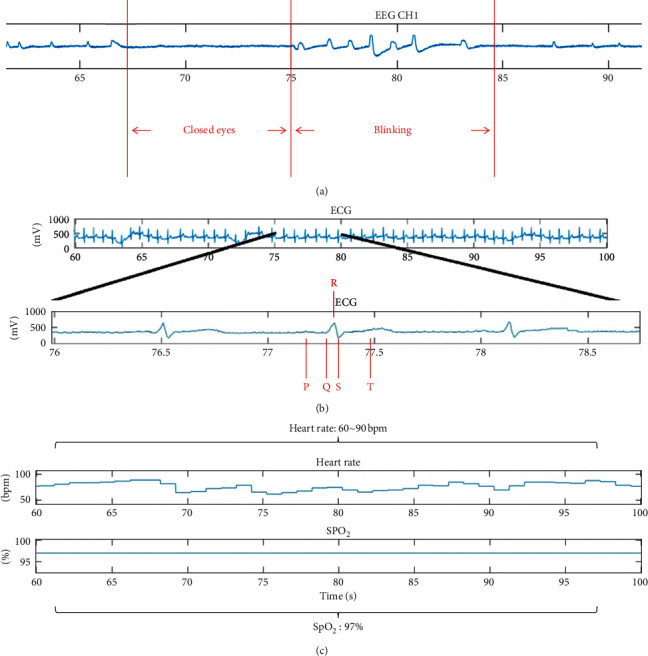
(a) Brain wave signals corresponding to closed eyes and blinking eyes. (b) Enlarged view of the ECG signals, showing that every complete cycle of the heart can be regarded as a complete PQRST wave. (c) HR and blood oxygen measurements.

In the integration interface test, the subject wears all the sensors of each module, including four EEG channels from BR8, three electrodes from AD8232, and one SpO_2_ channel from MAX30100 (Figure 5). Four EEG electrodes are placed on the forehead (Figures 5(a) and 5(b)), three ECG electrodes are placed on the chest (Figure 5(c)), and the subject's finger is placed on MAX30100 for the SpO2 measurement (Figure 5(c)). When the preparation procedure is finished, the subject begins to undergo measurement of the EEG, ECG, and SpO2 signals simultaneously. The results we obtained are presented in the integrated interface shown in Figure 5(d). The first to fourth channels are the signals received from the four electrodes of the EEG measurement. The fifth channel is the ECG signal, measured by the aforementioned three electrodes. The sixth and seventh channels are the HR and SpO_2_ signals. In the experiment, we ask the subject to close their eyes for approximately 10 seconds and then quickly blink for approximately 10 seconds. As shown, for approximately 67–75 seconds, the EEG signal has a gentle waveform, which represents the brain wave of the closed eye. Between 75 seconds and 85 seconds, each peak represents an eye blink (Figure 6(a)). Then, after enlarging the ECG signal, we observe that every complete cycle of the heart rhythm is a PQRST wave (Figure 6(b)). Finally, the HR and SpO2 values fall within the normal range (Figure 6(c)).

## 4. Conclusions

This study developed a multiple-biosignal recording system based on a modular design that integrates EEG, ECG, and SpO_2_ modules for medical applications. The data from these three modules are transmitted to the microcontroller unit, an Arduino board, through a designed logic level converter. The Arduino board uses COBS to simultaneously decode the data and send them together to a customized graphical user interface for near-real-time presentation in a MATLAB environment, which is capable of receiving and visualizing data in real time and providing data for offline analysis. This system passed all simulation signal tests and functioned as intended when applied to a real human being. The modular design can endow the integrated system with high flexibility and enable our EEG module to be successfully combined with other commercial measurement devices on a common Arduino platform. In the future, we will replace our microcontroller with a more advanced microcontroller (e.g., the Arduino Zero) to increase the speed of data transmission. This improvement can help to enable the measurement of different biosignals (e.g., electromyography) or channels of the EEG module in this system for complete monitoring of human health status. Moreover, Wi-Fi/Bluetooth will support mobile platforms to increase the convenience of usage and application. We anticipate this multiple-biosignal acquisition system to be used for various real-life medical applications, such as in emergency or ICU settings, and to improve human quality of life.

## Figures and Tables

**Table 1:** ADS1298 specification and specification of the active circuit.

*ADS1298:8-channel ADC*	
Resolution	24 bit/16 bit high resolution
Input channels	8 channels
Power/channel consumption	0.75 mW
Control interface	SPI

*Specification of active circuit*	
Signal	EEG
Input signal range	10 *µ*V–100 *µ*V
Gain	1357
Sampling rate (Hz)	512

**Table 2:** Example of COBS.

Unencoded data (hex)	Encoded with COBS (hex)
00	01 01 00
00 00	01 01 01 00
11 22 00 33	03 11 22 02 33 00
11 22 33 44	05 11 22 33 44 00
11 00 00 00	02 11 01 01 01 00

## Data Availability

Data will be provided on request through the corresponding author of this article.
